# Real-time Humidity Sensor Based on Microwave Resonator Coupled with PEDOT:PSS Conducting Polymer Film

**DOI:** 10.1038/s41598-017-18979-3

**Published:** 2018-01-11

**Authors:** Jin-Kwan Park, Tae-Gyu Kang, Byung-Hyun Kim, Hee-Jo Lee, Hyang Hee Choi, Jong-Gwan Yook

**Affiliations:** 1 Yonsei University, Department of Electrical and Electronic Engineering, Seoul, 120-749 Republic of Korea; 2 Daegu University, Department of Physics Education, Gyeongsan, Gyeongbuk, 705-714 Republic of Korea; 3 Yonsei University, Institute of Engineering Research, Seoul, 120-749 Republic of Korea

## Abstract

A real-time humidity sensor based on a microwave resonator coupled with a poly(3,4-ethylenedioxythiophene) polystyrene sulfonate (PEDOT:PSS) conducting polymer (CP) film is proposed in this paper. The resonator is patterned on a printed circuit board and is excited by electromagnetic field coupling. To enhance the sensitivity of the sensor, the CP film is located in the area with the strongest electric field in the resonator. To investigate the performance, the proposed sensor is placed alongside a reference sensor in a humidity chamber, and humidity is injected at room temperature. The experimental results indicate that the electrical properties of the resonator with the CP film, such as the transmission coefficient (*S*_21_) and resonance frequency, change with the relative humidity (RH). Specifically, as the RH changes from 5% to 80%, *S*_21_ and the resonance frequency change simultaneously. Moreover, the proposed sensor exhibits great repeatability in the middle of the sensing range, which is from 40% to 60% RH. Consequently, our resonator coupled with the CP film can be used as a real-time humidity-sensing device in the microwave range, where various radio-frequency devices are in use.

## Introduction

Humidity sensors have recently been receiving considerable attention due to their usefulness in various fields, such as agriculture, manufacturing, environmental sciences, medicine^1,2^, and so on. These humidity sensors should possess good performance characteristics, such as high accuracy, good repeatability, wide sensing range, fast response time, easy fabrication process, and low production cost. To satisfy these requirements, various sensing mechanisms and materials have been studied. The sensing mechanisms are classified based on the components used for the measurement. The first class includes optical fibre sensors^3–5^, which measure the reflectivity by adding a sensing layer to the end of an optical fibre. The second class includes sensors based on nanowires^6^ and bulk acoustic wave resonators^7^, which coat the sensing layers on the components and measure the resonance frequencies at very low frequencies. The third class measures the impedance change by depositing the sensing layer on interdigitated electrode (IDT)^8,9^. The fourth class measures the capacitance change of a dielectric membrane by filling the disk witg the sensing material^10,11^. The final class includes chemically sensitive field-effect transistor (ChemFET)^12,13^ sensors, which substitute the gate region of the transistor with the sensing material and measure the drain current. Various sensing materials such as carbon nanotubes^14,15^, crystals^16^, graphene^17–19^, ceramics^20,21^, silicon^22^, organic polymers^23,24^, and composite materials^25,26^, have been studied. However, despite these studies, more investigations are required to achieve high sensitivity, real-time sensing, easy fabrication, and great repeatability characteristics.

Among the sensing materials, poly(3,4-ethylenedioxythiophene) polystyrene sulfonate (PEDOT:PSS) has been receiving considerable attention for use in sensors because of its outstanding properties, such as high conductivity, good processability, low price, low redox potential, and ability to operate at room temperature^27–29^. Most previous studies that used PEDOT:PSS as an analyte focused on FETs^30,31^ or electrodes^32,33^ at the DC level or on a surface acoustic wave (SAW) filter^34^ at low frequencies. However, these methods could measure only one parameter, such as the current, resistance, or resonance frequency, to deduce the result. In addition, it was difficult to fabricate and enhance the sensitivity of FET and nanowire-based sensors, and it was difficult to integrate SAW filter-based sensors into a single integrated circuit (IC); it was also difficult to miniaturize the sensors.

Meanwhile, microwave resonators can measure two parameters, namely, the transmission coefficient passing through the resonator (*S*_21_) and the resonance frequency, to enhance the detection capacity. Moreover, their sizes can be reduced due to their high operating frequencies, and they can easily be linked with commercial mobile communication systems. Because of these advantages, microwave resonators have been applied in a variety of sensing applications, such as glucose measurement^35,36^, biomolecular sensing^37–39^, gas sensing^40,41^, wrist pulse detection^42–44^, vital sign monitoring^45–47^, and breast tumour detection^48,49^. Additionally, relative humidity detection using microwave components has also been studied^50,51^. In contrast to previous works, a PEDOT:PSS coupled microwave resonator is proposed in this paper. It is determined that by replacing a part of the conductor, the sensitivity of the sensor can be enhanced and its fabrication becomes easy. Moreover, the proposed sensor can be used in real time (<0.5 s) in cases where the relative humidity (RH) ranges from 5% to 80%, with excellent repeatability. When the RH changes from 5% to 80%, *S*_21_ and the resonance frequency are immediately changed by 0.18 dB and −35.4 MHz,respectively.

## Results

### Simulation of microwave resonator

**Figure 1:**
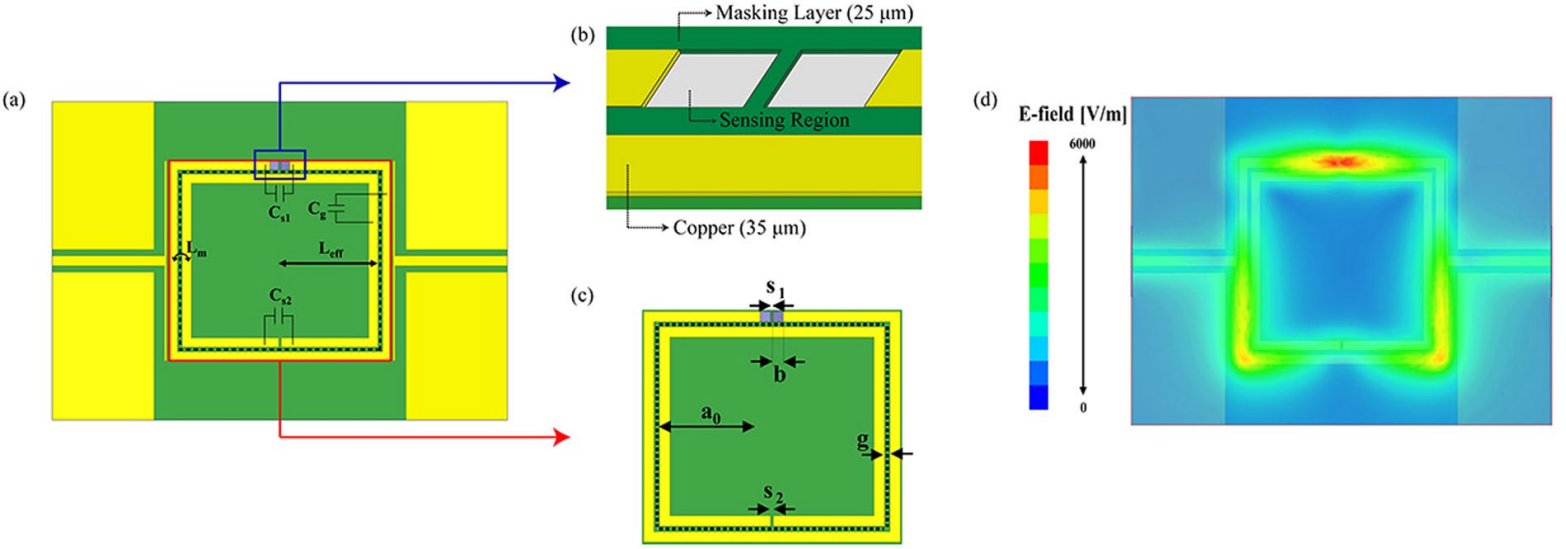
Design properties of the resonator and its simulation result. (**a**) Electric components of double split-ring resonator (DSRR). (**b**) Sensing region of CP film. (**c**) Dimensions of DSRR (a_0_ = 11.5 mm, b = 1 mm, d = 0.5 mm, g_1_ = 0.2 mm, and g_2_ = 0.3 mm). (**d**) Simulation result of E-field distribution (1.5 mm above the substrate).

In this work, we designed a microwave resonator, namely, a double split-ring resonator (DSRR), that is compatible with a CP film for a real-time humidity sensor. The operating frequency of the resonator was chosen to be 2.45 GHz, which is a specific frequency in the industrial-scientific-medical (ISM) band, for applications in wireless sensing platforms. The resonance frequency of the DSRR is generally determined using the following equation^52,53^:

1 $$\begin{array}{ll}{\omega }_{0}=\sqrt{\frac{1}{2{a}_{0}LC}} & \\ L={L}_{eff}+{L}_{m}, & C={C}_{S1}+{C}_{S2}+{C}_{g}\end{array}$$

where a_0_ is the distance between the centre of the square and the middle of the inner and outer squares, as indicated in Fig. 1c. L_*eff*_, L_*m*_, C_*S*1_, C_*S*2_, and C_g_ are the effective inductance obtained from a_0_, the mutual inductance between the two squares, the capacitance between the split of each ring, and the capacitance between the two rings, respectively, as illustrated in Fig. 1a.

To prepare the sample, the proposed sensor was fabricated on a printed circuit board (PCB) substrate, which was 0.762 mm thick with a dielectric constant (*ε*_*r*_) of 6.15 and loss tangent (tan*δ*) of 0.0028. After electroplating nickel (Ni) as an adhesion layer, the resonator with a copper pattern was coated with gold to prevent oxidation from humidity and then with a PEDOT:PSS CP film. To deposit the CP film at the specific site and to prevent overflow, a mask layer was added to the substrate, which covered all parts except for the copper line, and to the sensing region. This layer was to form a well structure, as shown in Fig. 1b. The mask layer was composed of photoimageable solder resist ink, which is widely used in the PCB process. Furthermore, to enhance the sensitivity, the sensing region was positioned between the outer electrodes, which is the region with the strongest electric field, as shown in Fig. 1d. This is because the material properties, namely, permittivity and conductivity, are dependent on the electric field, and the electric field effects can sensitively change the resonance frequency and the gain level of the transmission coefficient (*S*_21_). For this reason, when a CP film is exposed to humidity, its permittivity and conductivity change simultaneously. Consequently, the resonator with a CP film can exhibit real-time deviations in frequency and a gain level of *S*_21_. After selecting the sensing region, the CP film was deposited onto two squares with a side of length b and gap size of g_i_. The dimensions of the other resonator sections are presented in Fig. 1c. Here, the feed line had a characteristic impedance of 50 Ω to avoid an impedance mismatch with the measuring vector network analyser (VNA). The distance between the feed line and the resonator, which controlled the amount of signal coupling to the resonator, was determined to be 0.2 mm. Physically, as this spacing increases, the signal coupling to the resonator decreases as presented in supplementary information Fig. S1.Therefore, keeping this distance short provides an advantage to the sensor. A ground plane was added to both sides of the feed line to reduce the radiation losses, dispersion, and parasitic wave propagation in the resonator.

### Validation of the sensor

**Figure 2:**
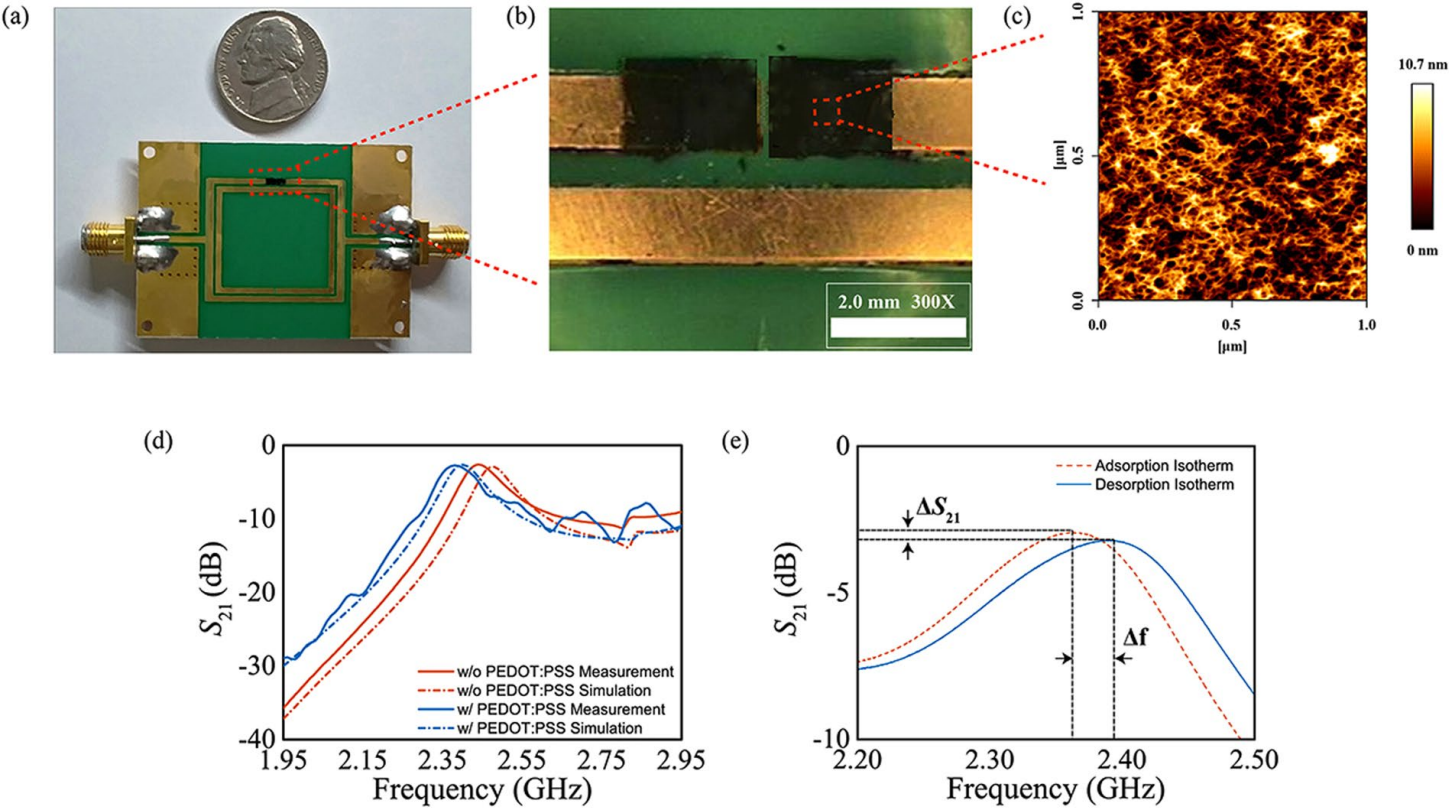
Characterization of the sensor. (**a**) Fabricated humidity sensor compared with a 5 cent US coin. (**b**) Optical microscope image of the sensor at the sensing region. (**c**) 2D AFM images of the surface of PEDOT:PSS deposited on the substrate. (**d**) *S*_21_ frequency response of DSRR. (**e**) Isotherm characteristics of the sensor versus RH adsorption and desorption.

The proposed RH sensor was fabricated using the well-defined PCB technology, as illustrated in Fig. 2a. To connect the sensor with the VNA, sub-miniature version A (SMA) connectors were mounted on the input and output feed lines. CP film deposition was confirmed using an optical microscope at 300x magnification, as shown in Fig. 2b. Due to the manual deposition of PEDOT:PSS, an imperfect square shape was obtained; specifically, near the gap region, the PEDOT:PSS layer was also detached when the polyimide film was removed. Therefore, the dimensions of the sensing region were deformed and caused ripples in the characteristics of the sensor in the frequency range of 2.55 GHz to 2.95 GHz, as shown in Fig. 2d. Atomic force microscopy (AFM) images of the deposited CP film surface are shown in Fig. 2c. The AFM images show that the PEDOT:PSS is evenly deposited on the substrate and that the average roughness of the PEDOT:PSS is 1.933 nm. Moreover, morphologies of the PEDOT:PSS observed using a the scanning electron microscope (SEM) are shown supplementary information Fig. S2. X-ray photoelectron spectroscopy (XPS) survey spectra of deposited PEDOT:PSS film are presented in supplementary information Fig. S3. A comparison of the frequency responses of the bare resonator and the PEDOT:PSS coupled resonator is presented in Fig. 2d. A simulation was conducted using a three-dimensional full-wave electromagnetic field solver, and measurements were conducted using the VNA. The microwave properties of the circuit modelling PEDOT:PSS used in the simulation are presented in supplementary information S4. After depositing the CP film on the resonator, the resonance frequency was shifted from 2.45 GHz to 2.4 GHz. This is because the film is a conductive material, and the deposition of the film caused an increase in the capacitance (C_*S*1_). Thus, the resonance frequency was slightly shifted to a lower frequency region. Figure 2e shows the measured isotherm characteristics of the sensor before and after the introduction of humidity. After the humidity reacted with the PEDOT:PSS CP film and was adsorbed by the film, the magnitudes of both *S*_21_ and the resonance frequency were affected. The gain level of *S*_21_ increased, and the resonance frequency slightly shifted towards a lower frequency.

**Figure 3:**
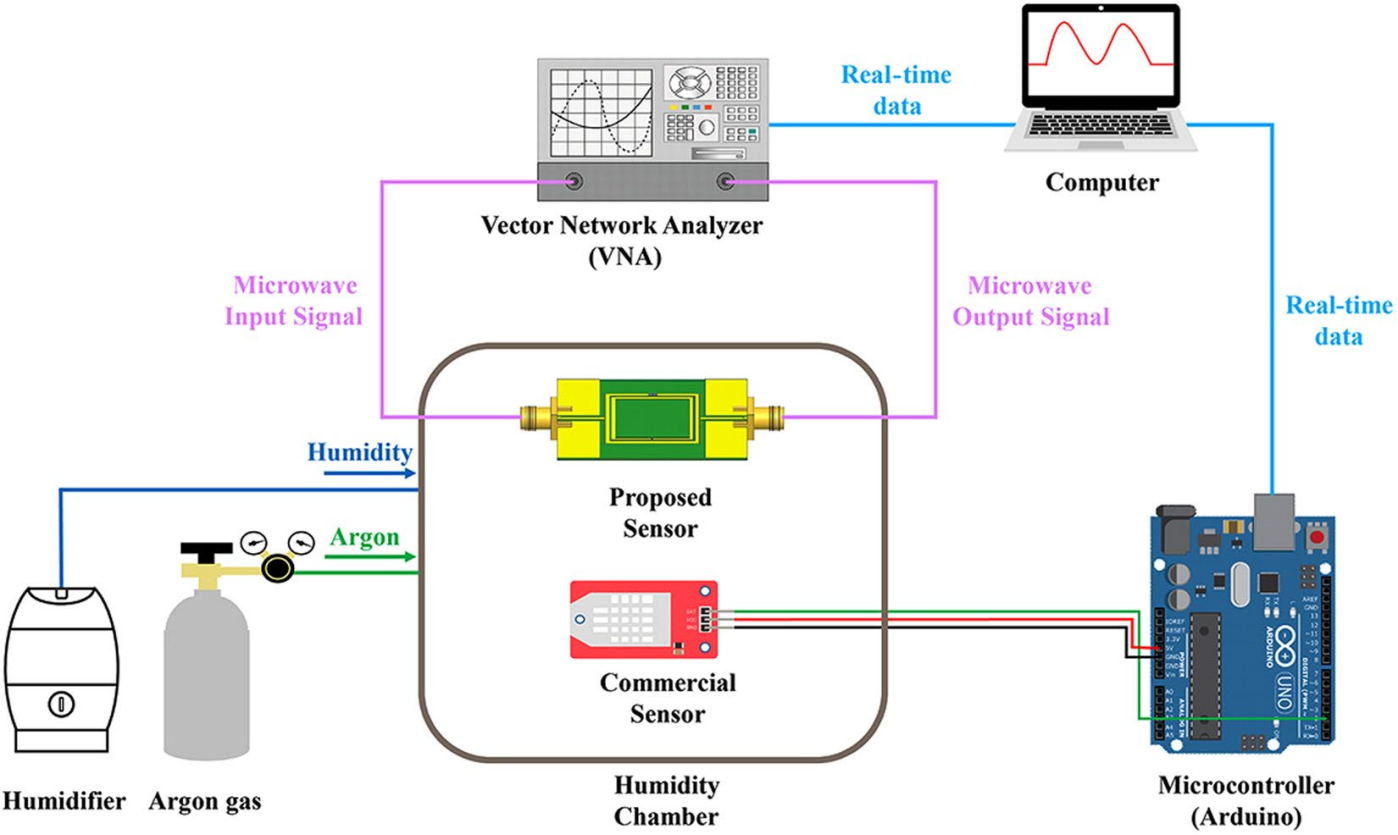
Experimental set up for relative humidity control in humidity chamber and testing the performance of the sensor.

The sensing response of the sensor was verified in a custom-made acrylic humidity chamber. The experimental setup for the fabricated sensor is illustrated in Fig. 3. A DSRR was placed along with a commercial sensor inside the humidity chamber, and the performance of the sensor was evaluated by comparing with the commercial sensor. The frequency response of the DSRR was measured using a VNA, and the measured data were sent to a computer every 0.5 s. The commercial sensor was connected to a micro-controller board, and the board also sent data to the computer every 0.5 s. The RH inside the chamber was increased by the humidifier and decreased by pure argon (Ar), where the controllable RH range is from 5% to 80%.

To analyse the performance of the proposed humidity sensor quantitatively, the following parameters are defined:

2 $$\begin{array}{cc}{\rm{\Delta }}{S}_{21}={S}_{\mathrm{21,}Humidity}-{S}_{\mathrm{21,}Ref} & (dB)\\ {\rm{\Delta }}F={F}_{Humidity}-{F}_{Ref} & (MHz)\end{array}$$

where *S*_21_ is the transmission coefficient at the resonance frequency. *S*_21,*Humidity*_ and *F*_*Humidity*_ are the values when the sensor reacts to humidity. *S*_21_,_*Ref*_ and *F*_*Ref*_ are the reference values when humidity is very low (3%). Here, Δ*S*_21_ and Δ*F* are the differences in the transmission coefficient and resonance frequency, respectively.

**Figure 4:**
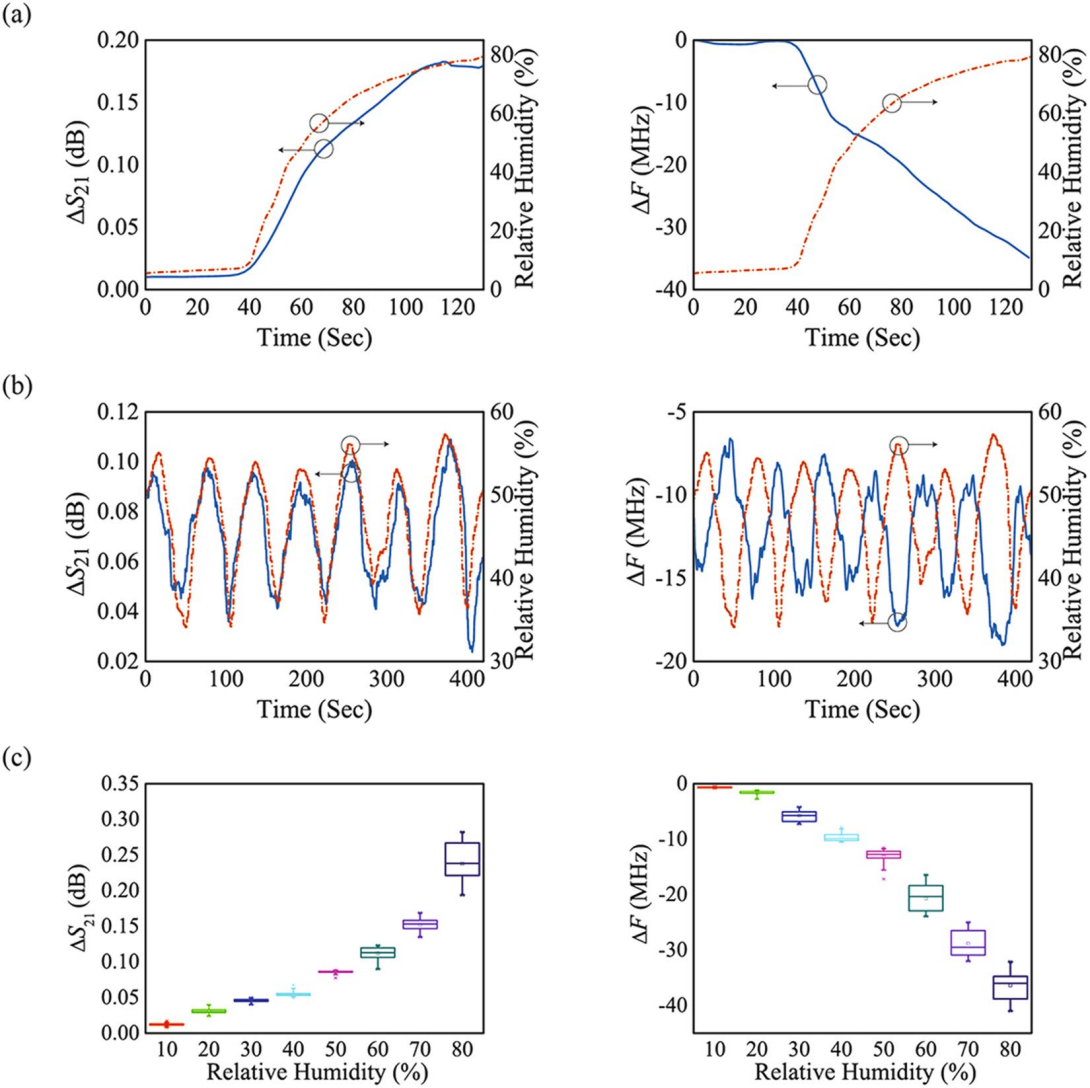
Measured results of Δ*S*_21_ and Δ*F* deviation as the RH changes. (**a**) Time-domain results of verifying the operating range. (**b**) Time-domain results of verifying repeatability. (**c**) Statistical results of repeated measurements.

The performance of the sensor was tested using two methods. First, the available operating range of the sensor was evaluated. The operating range was verified by venting most of the humidity in the chamber using argon (Ar) and injecting humidity into the dehumidified custom-made chamber at room temperature (RT). The RH was increased using a humidifier for 130 s until it reached 80%. Δ*S*_21_ and Δ*F* were tracked in the time domain as functions of the RH values. As the RH increased, Δ*S*_21_ increased by 0.18 dB and Δ*F* decreased by −35.4 MHz, as shown in Fig. 4a. It was found that the changes in *S*_21_ and the resonance frequency exhibited opposite trends with the RH level and that the microwave sensor was suitable for a real-time humidity sensor when the RH was in the range between 5% and 80%.

Next, the repeatability of the sensor was tested by controlling the RH in the middle of sensor’s operating range, which is from 40% to 60%. The test consisted of seven cycles, with each cycle lasting 60 s. In each cycle, half of the cycle was designated for the injection of humidity, while the other half was for ventilation. As the RH changed, Δ*S*_21_ changed from 0.04 dB to 0.1 dB, while Δ*F* shifted from −7 MHz to −15 MHz, as shown in Fig. 4b. The result showed that the proposed sensor responded to humidity in real time and that it had an outstanding repeatable response.

In addition to the repeatability test, ten samples with separately deposited CP films were prepared for testing, and each sample was measured three times. The statistical distributions of the measured data are shown in the box plot in Fig. 4c and summarized in Table 1. As the RH increased, Δ*S*_21_ and Δ*F* changed quite rapidly, and the difference between each sample (i.e., the standard deviation) increased because of the PEDOT:PSS saturation^54^. Nevertheless, it was clearly observed that the proposed microwave sensor could be used to detect RH by measuring the changes in *S*_21_ and those in the resonance frequency. Comparisons with other microwave-based RH sensors are shown in supplementary information Table S1.

**Table 1:** Mean values of Δ*S*_21_ and Δ*F*.

Relative Humidity (%)	Δ*S*_21_ (dB)	Δ*F* (MHz)
10	0.013	−0.572
20	0.034	−1.68
30	0.050	−4.206
40	0.068	−8.153
50	0.087	−12.43
60	0.118	−25.686
70	0.164	−34.27
80	0.267	−42.66

## Discussion

A chemical reaction between the PEDOT:PSS CP film and humidity can clearly be observed via the microwave electrical properties, *S*_21_ and the resonance frequency. The *S*_21_ is related to the conductivity of the PEDOT:PSS CP film, which is inversely proportional to the square of the ohmic loss. Meanwhile, the resonance frequency is related to the capacitance between the PEDOT:PSS CP films (C_*S*1_), which is inversely proportional to the square of the resonance frequency. Moreover, the changes in the conductivity and capacitance are independent, meaning that they can provide twice the amount of information.

The chemical reaction can be verified in two ways. First, the changes in capacitance can be identified by measuring the change in the resonance frequency. This variation is a consequence of the deposited film deformation and the effective permittivity change. As the RH increases, water molecules around the PEDOT:PSS are accumulated and capillary action occurs. This fills the intergrain crevices of PEDOT:PSS with H_2_O. This process causes swelling of the film, which subsequently increases the film size^55^. Additionally, the adsorption of H_2_O increases the effective permittivity because H_2_O has high permittivity (*ε*_*r*_ = 80)^56^. Because the capacitance is proportional to the areas of the two conductors and the permittivity, an enhancement in the capacitance can be observed. Consequently, the resonance frequency shifts to a lower frequency region, which is consistent with the result in Fig. 4.

**Figure 5:**
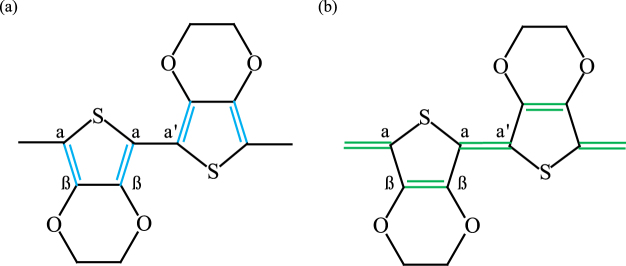
Chemical structure of PEDOT. (**a**) Benzoid structure. (**b**) Quinoid structure.

Second, the changes in the conductivity can be deduced by measuring *S*_21_. This change is a result of the chemical structure of PEDOT. PEDOT:PSS is a conjugated conducting polymer, which is composed of poly(3,4-ethylenedioxythiophene) (PEDOT) and polystyrene sulfonate (PSS). PEDOT:PSS has a two-chemical conformation with benzoid and quinoid structures, which are two resonance structures of PEDOT. There are two conjugated *π* electrons on the C_*α*_=C_*β*_ bond in the benzoid structure, and there are no conjugated *π* electrons on the C_*α*_-C_*β*_ bond in the quinoid structure, as illustrated in Fig. 5. Thus, the quinoid structure has delocalized ions, and these ions make the quinoid structure more conductive than the benzoid structure. Without additives, both benzoid and quinoid structures are present in PEDOT:PSS. However, adding organic compounds with certain chemical conditions transforms the benzoid structure into the quinoid structure. The additives must have two or more polar groups in a molecule, and they must enable the formation of hydrogen bonds with the PSS of PEDOT:PSS^57,58^. Thus, when PEDOT:PSS reacts with humidity, an enhancement in conductivity can be observed. This increases *S*_21_, which is consistent with the result in Fig. 4.

In summary, a humidity sensor device based on a microwave resonator coupled with a PEDOT:PSS CP film was successfully demonstrated. The performance of the proposed sensor was verified by comparing it with a commercial sensor in two aspects: operating range and repeatability. As the RH changed, both *S*_21_ and the resonance frequency varied faster than 0.5 s. Note that similar results were obtained for different samples. Our results demonstrated that the microwave resonator with a CP film was suitable for humidity monitoring at room temperature in real time.

## Methods

### Sensing Material Synthesis and Deposition

Poly(3,4-ethylenedioxythiophene) polystyrene sulfonate (PEDOT:PSS) solution (Clevios PH 1000) was purchased from Heraeus. The solid content was 1.1 wt%, and the weight ratio of PEDOT to PSS was 1:2.5. Dimethyl sulfoxide (DMSO, 99%) was purchased from Samchun Pure Chemicals (S. Korea). To enhance the electrical conductivity of the PEDOT:PSS film, the typical DMSO doping procedure was followed. DMSO was added to the aqueous PEDOT:PSS solution (5.0 v/v%) and stirred gently at room temperature. Then, the solution was filtered using a syringe filter (0.45 μm pore-size Nylon membrane).

The PEDOT:PSS films were deposited as follows. First, polyimide tape was attached to the surface of the sensor as a shield to prevent the analyte region from overflowing. Although the substrate was inherently hydrophobic, the surface could be temporarily rendered hydrophilic upon exposure to oxygen plasma for adhesion improvement. Then, the polymer solution was dropped onto the substrate and bar coated to stabilize the roughness.

### Equipment

The 2D surface view of the deposited film was observed by AFM (Nanowizard I, JPK instrument). The morphologies of the deposited film were observed by SEM (JSM-7001F, JEOL). The XPS survey spectra of the deposited PEDOT:PSS film were obtained using an XPS (K-alpha, Thermo Scientific). The characteristics of the sensor were measured using a VNA (E5071B, Agilent), which was controlled by LabView (NI) through a general-purpose interface bus (GPIB) cable. A commercial reference sensor (DHT22) was connected to a microcontroller board (Arduino), which interfaced the sensor with a computer. The DHT22 is a capacitive humidity sensor, whose operating range is from 0% to 100% and response time is 2 s. The resolution is 0.1% RH, and the accuracy is ±2 RH.

## Electronic supplementary material

Supplementary information
