# Supplementary material for: Real-time Humidity Sensor Based on Microwave Resonator Coupled with PEDOT:PSS Conducting Polymer Film
Source: Sci Rep. 2018 Jan 11;8:439. doi: 10.1038/s41598-017-18979-3 (PMC5764980; doi:10.1038/s41598-017-18979-3)
Supplement: Supplementary file 1 — Supplementary information [file 41598_2017_18979_MOESM1_ESM.pdf]

SUPPLEMENTARY INFORMATION

Real-time Humidity Sensor Based on Microwave Resonator Coupled with PEDOT:PSS Conducting Polymer Film

Jin-Kwan Park[1], Tae-Gyu Kang[1], Byung-Hyun Kim[1], Hee-Jo Lee[2], Hyang Hee Choi[3] Jong-Gwan Yook[1,*]

[1] Yonsei University, Department of Electrical and Electronic Engineering, Seoul, 120-749, Republic of Korea

[2] Daegu University, Department of Physics Education, Daegu, 705-714, Republic of Korea

[3] Yonsei University, Institute of Engineering Research, Seoul, 120-749, Republic of Korea

*jgyook@yonsei.ac.kr

1. Signal level coupling to the resonator

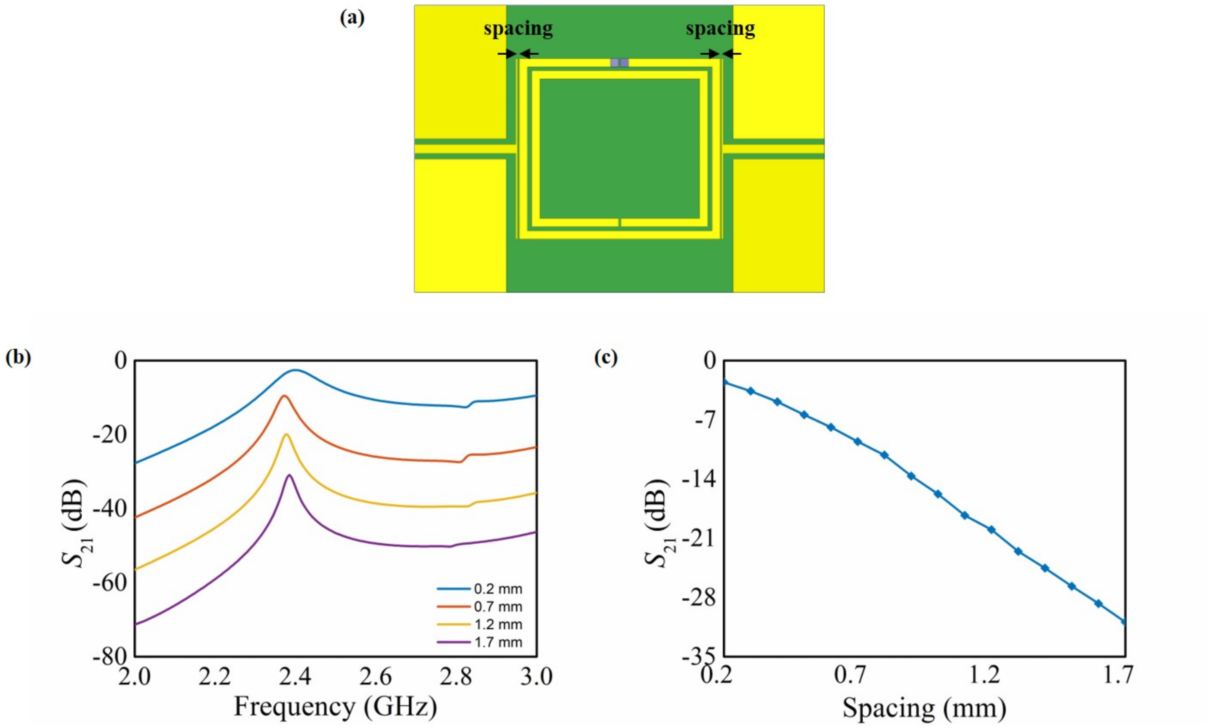

Figure S1. (a) The spacing between the signal line and the resonator. (b) Transmission coefficient of the resonator versus spacing (from 0.2 mm to 0.5 mm intervals). (c) Transmission coefficient of the resonator versus spacing (from 0.2mm to 0.1 mm intervals) at the resonance frequency.

2. Morphologies of the deposited PEDOT:PSS

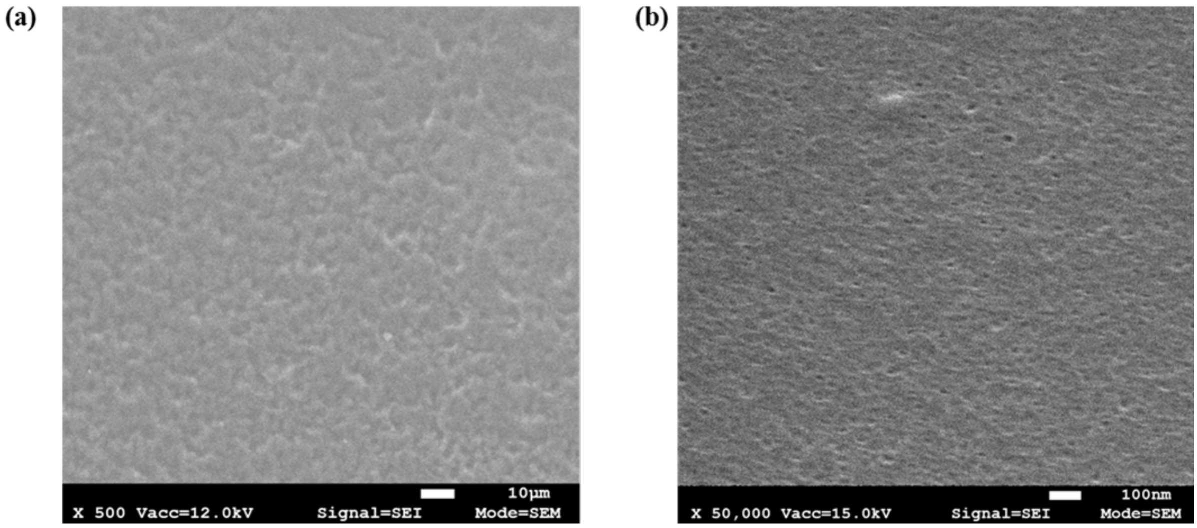

Figure S2. SEM images of the deposited film (a) Working distance = 8mm, acceleration voltage = 12.0 kV, magnification of 500. (b) Working distance = 8mm, acceleration voltage = 15.0 kV, magnification of 50,000.

3. XPS survey spectra of deposited PEDOT:PSS film

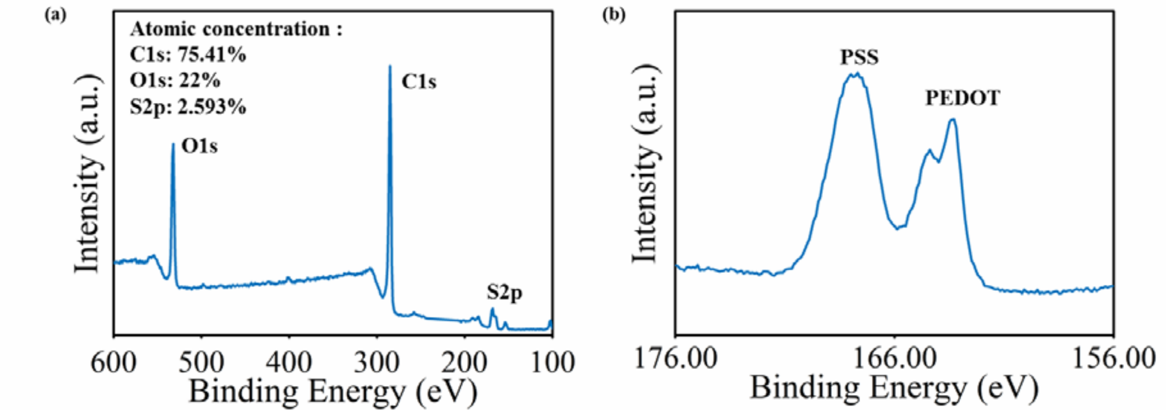

Figure S3 XPS spectra of deposited PEDOT:PSS film: (a) XPS general scan (b) Sulphur S(2p) (PEDOT = 163.4, 164.4 eV, PSS = 167.7 eV).

4. Verifying microwave characteristics of PEDOT:PSS[1]

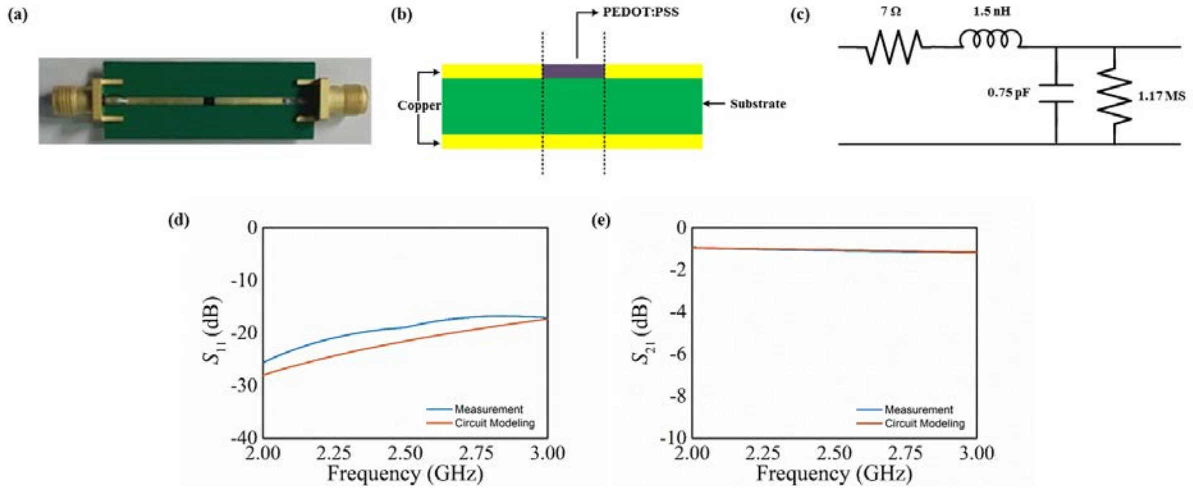

Figure S4. (a) The fabricated sample for verifying the microwave characteristics of PEDOT:PSS. (b) Side view of PEDOT:PSS-doped transmission line. (c) Circuit modelling of PEDOT:PSS-doped transmission line. (d) Reflection coefficients of measured and modelled PEDOT:PSS-doped transmission line. (e) Transmission coefficients of measured and modelled PEDOT:PSS-doped transmission line.

5. Comparison of RH sensors based on microwave components

Table S1. Comparison of RH sensors based on microwave components

Component	Sensing Principle	RH Range	Sensing material	Sensitivity	Repeatability	Response Time	Ref.
Oscillator	Frequency shift	0-40%	Polyimide	5 kHz/RH	X	68 sec	[2]
Slotline ring resonator	Power level, Frequency shift	30-90%	Polyimide	4.95 mdB/RH 181 kHz/RH	X	X	[3]
Antenna				5.50 mdB/RH -108 kHz/RH			
RFID tag	Frequency shift	25-90%	Polyimide	108 kHz/RH	X	X	[4]
RFID tag	Frequency shift	11.3-100%	Kapton HN	171.4 kHz/RH	X	X	[5]
Stepped impedance resonator	Frequency shift	65-80%	Kapton HN Polyimide	200 kHz/RH	X	X	[6]
H-slot antenna	Power level	50-100%	PEDOT:PSS	0.2 dB/RH	O	X	[7]
Substrate integrated resonator	Frequency shift	0-80%	X	101 kHz/RH	X	X	[8]
				9.35 kHz/RH			
Double split-ring resonator	Power level, Frequency shift	5-80%	PEDOT:PSS	2.4 mdB/RH 0.47 MHz/RH	O	< 0.5 Sec	This work

References

[1] K.C. Gupta, R. Garg, R. Chadha, Computer aided design of microwave circuits, *NASA STI/Recon Technical Report A* **82**, (1981).

[2] Bernou, C., Rebière, D. & Pistré, J. Microwave sensors: a new sensing principle. Application to humidity detection. *Sensors Actuators, B Chem*. **68**, 88–93 (2000).

[3] Kim, Y. H., Jang, K., Yoon, Y. J. & Kim, Y. J. A novel relative humidity sensor based on microwave and a customized polymeric film. *Sensors Actuators, B Chem*. **117**, 315–322 (2006).

[4] Chang, K., Kim, Y.H., Kim, Y.J. & Yoon, Y.J. Functional antenna integrated with

relative humidity sensor using synthesized polyimide for passive RFID sensing, *Electron Lett*. **43**, 7–8 (2007).

[5] Virtanen, J., Ukkonen, L., Björninen, T., Elsherbeni, A. Z. & Sydänheimo, L. Inkjet-printed humidity sensor for passive UHF RFID systems. *IEEE Trans. Instrum. Meas*. **60**, 2768–2777 (2011).

[6] Amin, E. M. & Karmakar, N. C. Development of a low cost printable humidity sensor for chipless RFID technology. *Proc. IEEE Int. Conf. RFID-Technol. Appl. (RFID-TA)*,165–170 (2012).

[7] Manzari, S. et al. Humidity sensing by polymer-loaded UHF RFID antennas. *IEEE Sens. J*. **12**, 2851–2858 (2012).

[8] El Matbouly, H., Boubekeur, N. & Domingue, F. Passive Microwave Substrate Integrated Cavity Resonator for Humidity Sensing. *IEEE Trans. Microw. Theory Tech*. **63**, 4150–4156 (2015).
